# A light-induced shortcut in the planktonic microbial loop

**DOI:** 10.1038/srep29286

**Published:** 2016-07-11

**Authors:** Robert Ptacnik, Ana Gomes, Sarah-Jeanne Royer, Stella A. Berger, Albert Calbet, Jens C. Nejstgaard, Josep M. Gasol, Stamatina Isari, Stefanie D. Moorthi, Radka Ptacnikova, Maren Striebel, Andrey F. Sazhin, Tatiana M. Tsagaraki, Soultana Zervoudaki, Kristi Altoja, Panagiotis D. Dimitriou, Peeter Laas, Ayse Gazihan, Rodrigo A. Martínez, Stefanie Schabhüttl, Ioulia Santi, Despoina Sousoni, Paraskevi Pitta

**Affiliations:** 1 ICBM, University of Oldenburg, Schleusenstrasse 1, DE-26382 Wilhelmshaven, Oldenburg, Germany; 2 WasserCluster Lunz - Biologische Station GmbH, Dr Carl Kupelwieser Promenade 5, A-3293 Lunz am See, Austria; 3 Institut de Ciències del Mar, CSIC, Pg Marítim de la Barceloneta, 37-49, 08003 Barcelona, Catalunya, Spain; 4 Department of Biology, University of Bergen, P.O. Box 7803, 5020 Bergen, Norway; 5 Leibniz Institute of Freshwater Ecology and Inland Fisheries, Dep. 3, Experimental Limnology, Alte Fischerhütte 2, 16775 Stechlin, Germany; 6 Red Sea Research Center, Biological and Environmental Sciences and Engineering Division, King Abdullah University of Science and Technology, 23955-6900 Thuwal, Kingdom of Saudi Arabia; 7 European Institute for Marine Studies, Technopole Brest-Iroise, Plouzane, France; 8 P.P. Shirshov Institute of Oceanology RAS, 36 Nahimovski prospect, 117997 Moscow, Russia; 9 Institute of Oceanography, Hellenic Centre for Marine Research, P.O. Box 2214, 71003 Heraklion, Crete, Greece; 10 Institute of Oceanography, Hellenic Centre for Marine Research, P.O. Box 712, 19013 Anavyssos, Greece; 11 Marine Systems Institute at Tallinn University of Technology, Akadeemia Rd. 15A, 12618 Tallinn, Estonia; 12 Department of Biology, University of Crete, Voutes University Campus, 70013 Heraklion, Crete, Greece; 13 Middle East Technical University, Institute of Marine Sciences, P.O. Box 28, 33731, Erdemli, Mersin, Turkey; 14 Department of Limnology, University of Vienna, Althanstrasse 14, 1090, Vienna, AT.

## Abstract

Mixotrophs combine photosynthesis with phagotrophy to cover their demands in energy and essential nutrients. This gives them a competitive advantage under oligotropihc conditions, where nutrients and bacteria concentrations are low. As the advantage for the mixotroph depends on light, the competition between mixo- and heterotrophic bacterivores should be regulated by light. To test this hypothesis, we incubated natural plankton from the ultra-oligotrophic Eastern Mediterranean in a set of mesocosms maintained at 4 light levels spanning a 10-fold light gradient. Picoplankton (heterotrophic bacteria (HB), pico-sized cyanobacteria, and small-sized flagellates) showed the fastest and most marked response to light, with pronounced predator-prey cycles, in the high-light treatments. Albeit cell specific activity of heterotrophic bacteria was constant across the light gradient, bacterial abundances exhibited an inverse relationship with light. This pattern was explained by light-induced top-down control of HB by bacterivorous phototrophic eukaryotes (PE), which was evidenced by a significant inverse relationship between HB net growth rate and PE abundances. Our results show that light mediates the impact of mixotrophic bacterivores. As mixo- and heterotrophs differ in the way they remineralize nutrients, these results have far-reaching implications for how nutrient cycling is affected by light.

In plankton ecology, mixotrophy is defined as the combined capacity for photosynthesis and phagotrophy in one protist. For a long time, mixotrophy was assumed to be an exception in the phytoplankton, largely composed of photoautotrophic organisms. We now know that phagotrophy is found in the majority of phylogenetic branches which comprise the ‘phytoplankton’, including representatives of dinoflagellate alveolates, chrysophycean, raphidophycean, tribophycean ochristans, cryptophycean cryptophytes and prymnesiophycean haptophytes^1^,^2^. Likewise, a considerable number of ciliates contains chloroplasts or symbiontic algae^3^ whereas only diatoms and chlorophytes are at present considered strictly photoautotrophic. The increased awareness of phagotrophy in the phytoplankton erases the traditional dichotomy which divided protists into separated algae and protozoa^4^.

An increasing number of recent studies point out the prevalence of mixotrophic phytoplankton in natural communities^5^,^6^. Especially small-sized phototrophic flagellates have been shown to dominate bacterivory in the surface waters of oligotrophic seas^5^,^6^,^7^. The occurrence of mixotrophs under these conditions is taken to indicate that they have an advantage over photoautotrophic phytoplankton by obtaining limiting nutrients through phagotrophy, much in the way carnivorous plants and stony corals thrive in oligotrophic environments due to the combination of photosynthesis with heterotrophic ingestion^2^. This recent appreciation of mixotrophy in the phytoplankton comes along with the detection of a hitherto overlooked phylogenetic diversity in the size range <1–3 μm by molecular analyses^8^. Lineages with phagotrophic abilities like chrysophytes and haptophytes are particularly abundant in this recently appreciated biota^9^,^10^,^11^.

The advantage of mixotrophs under conditions of nutrient limitation seems obvious – by ingesting other algae and bacteria, a mixotrophic protist increases its gain in limiting nutrients, especially nitrogen, phosphorus and iron (e.g.^12^,^13^,^14^). This holds especially true for mixotrophic bacterivores. Bacteria have the highest surface-to-volume ratio among osmothrophic plankton, which gives them the highest affinity to limiting nutrients. In addition, heterotrophic bacteria are characterized by low C:N and C:P ratios^15^, making them a particularly attractive prey for mixotrophs which may cover their energy demand by photosynthesis but their nutrient requirements by phagotrophy. Bacterivory therefore represents an alternative access to nutrients when dissolved fractions are depleted.

Being a ‘jack of all trades’ implies costs, as mixotrophs must maintain both cellular machineries for photosynthesis (chloroplasts) and phagotrophy (structures for uptake and digestion, like e.g. haptonema and vacuole)^16^,^17^. Necessarily, a generalist like a mixotroph must be inferior in some way compared to more specialized competitors. The trade-off between nutrition modes is illustrated by comparing the maximum growth rates of related species being either a heterotroph or a mixotroph, as done by Rothhaupt^18^ with two closely related chrysophytes: *Spumella sp*. (heterotrophic; μmax = 2.4) and *Ochromonas sp*. (mixotropic; μmax = 0.9)^18^. Likewise, the metabolic rates of heterotrophic dinoflagellates commonly exceed those of mixotrophic representatives^19^. The comparison of maximum growth rates between mixo- and photoautotrophs is more difficult, as the precise mode of nutrition is unclear for many naturally abundant phototrophic flagellates. However, a large compilation of freshwater phytoplankton traits derived from observational data suggests that the growth rates of strictly photoautotrophic representatives (chlorophyceae: *Scenedesmus, Chlamydomonas*; bacillariophyceae: *Fragilaria, Cyclotella*) clearly exceed those of typical mixotrophs (chrysophyceae: *Dinobryon, Ochromonas*; haptophyta: *Chrysochromulina*; dinophyta: *Peridinium, Gymnodinium*; Table S1 in^20^). From that it appears that the niche of mixotrophs is mostly defined by maintaining positive growth at simultaneously low prey and nutrient concentrations. In such situations, they can successfully invade communities containing heterotrophic and photoautotrophic competitors^21^,^22^.

The advantage of mixotrophs in terms of sustaining positive growth at low prey and nutrient concentrations should be bound to the availability of light^23^,^24^. However, a rigorous test on the role of light as a key factor mediating the success of mixotrophic bacterivores in oceanic waters is missing. Our aim here is to test the role of light as a determining factor for the relative importance of heterotrophic and mixotrophic bacterivores in an oligotrophic oceanic system. Considering the traits of mixotrophic and heterotrophic bacterivores that are outlined above, we derive two essential predictions for how light should affect the dynamics of a microbial food web.

1. Light mediates the competitive success of mixotrophic vs. heterotrophic grazers for shared prey, with mixotrophs being favored by high light. We therefore expect the ratio mixotrophs: heterotrophs among protist consumers to increase with light.
2. We expect that mixotrophs may reduce bacterial abundances when light is saturating. Thus, abundances of bacteria would be expected to be inversely related to light levels.

To test these hypotheses, we conducted a mesocosm experiment using a natural plankton community from the ultra-oligotrophic Eastern Mediterranean Sea off Crete. We exposed natural seawater including micro- and mesozooplankton to four light levels spanning a 10-fold gradient in duplicate mesocosms. In the present analysis, we focus on the dynamics at the base of the microbial food web. Since the smallest organisms, such as heterotrophic bacteria, pico-cyanobacteria and their smallest grazers have the highest metabolic rates, we expected that they would show the most immediate numerical response to the experimental light manipulation, which lasted for nine days. Results concerning the larger protistan grazers and their interaction with mesozooplankton were presented in previous papers^25^,^26^.

## Materials and Methods

### Experimental setup

We carried out a mesocosm experiment in the mesocosm facility of the Hellenic Centre for Marine Research (HCMR) near Heraklion, Crete, in September 2010.

On two consecutive days, altogether 22 m^3^ of seawater from 10 m depth were taken 0.5 nautical miles off Heraklion, Greece, with a centrifugal pump. Acid washed 1 m^3^ containers were filled and transported to the experimental site, where the water was poured into the mesocosms by gravity. The volume of each container was split evenly among all 8 mesocosms. The mesocosms were constantly shaded by opaque covers and kept at *in situ* temperature during this period. On the evening of the second day, the filling was finalized and the mixing was started. Lids for manipulation of light were installed in the darkness after filling, so that the experimental manipulation started the following morning (day 0). The experiment was run for 9 days, including day 0.

### Mesocosm design

Each mesocosm was made of an inner transparent polyethylene bag, with a diameter of 132 cm and a depth of 2.5 m filled to a final volume of 2.8 m^3^. The inner transparent mesocosm wall was surrounded by a double layer of 0.1 mm thick plastic foliage with a black and a white side (Agrolin White/Black; Achaika Plastics, Athens). This double outer layer served as optical isolation and was mounted with the black sides together and white sides facing outwards. The outer white layer maximized reflection and thus optical isolation from the surrounding tank, while the inner white side reflected light inside the mesocosms, ensuring a moderate vertical light gradient. The eight mesocosms were suspended inside a concrete outdoor water basin (app. 5 × 15 m, 5 m deep). The basin was filled with brackish water from a well and was continuously replenished at a low rate to maintain a stable temperature close to *in situ* conditions.

**Figure 1:**
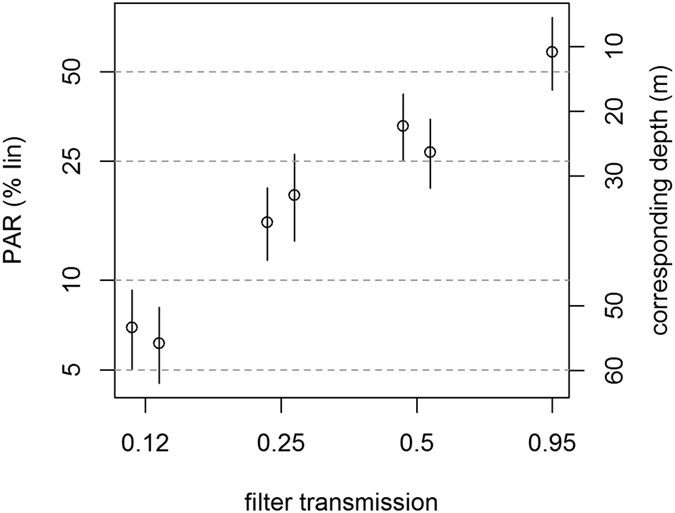
Light regime in the experiment. The left y-axis gives average (Imix) and range (error bars) of light intensities proportional to incoming light (Iin) as a function of the optical filters used. The right axis gives the corresponding optical depth in the Eastern Mediterranean, assuming an extinction coefficient of 0.05 m^−1^.

A four level light gradient was installed using neutral density filters (Lee Filters, UK). Light reduction was created by fixing filter foil onto a conical grid that was mounted on the top of each mesocosm so that light entered the mesocosms only through the filter foil. Hoods were firmly fixed, and we did not remove them throughout the experiment. The filters attenuated approximately 5, 50, 75 and 87 percent of the incoming natural light, resulting in an approximately logarithmic series of light intensities (Fig. 1; filter specifications: 130 clear, 95% transmission; 0.3ND, 0.6ND and 0.9ND with each 50, 25 and 12.5% transmission, respectively; Lee Filters, UK). Transmission for UV-B-radiation was <5% for all filters, while reduction of UV-A was similar to that of visible light.

The water inside each mesocosm was gently mixed by pumping air (app. 1 bubble per second) to the lower end of a plastic pipe (length 160 cm, 4 cm diameter). The pipe was suspended vertically in the mesocosm by a line, with its upper end reaching just below the water surface. The bubbling created a constant, gentle water flow from the deeper part of the mesocosm to the surface, ensuring continuous mixing of the mesocosms.

We measured vertical light profiles (PAR) in 0.5 m steps in all light level treatments using a spherical light sensor (LI-139SA, Licor NE, USA), with simultaneous measurements outside the mesocosm (flat quantum sensor LI-190SA, Licor, NE, USA). Based on these data, we calculated the attenuation caused by the optical hoods plus the vertical water column. Average light intensity (*I*_*mix*_) across the water column was then estimated based on standard formula^27^.

During sampling, water was collected in 10 L light-protected containers with opaque silicon tubing. The water level of the basin in which the mesocosms were suspended in was approx. 1 m above the surrounding working platform. This allowed taking samples by gravity.

### Nutrient measurements

Dissolved inorganic nutrients (PO_4_, NH_4_, NO_2_, NO_3_, SiO_2_) were initially measured daily, and every other day from day 3 onwards. Phosphate, silicate, nitrite and nitrate were analysed according to^28^ and ammonium according to^29^.

### Phosphorus-uptake

P-uptake was measured daily by incubating water samples with ^33^P. Different size-fractions of the particulate fraction (0.2–0.6 μm, >0.6–2 μm, >2–10 μm, >10 μm) were subsequently radio-assayed. Details are given here^30^.

### Heterotrophic bacterial activity

Bacterial activity was estimated daily as ^3^H-leucine incorporation rate into trichloroacetic acid (TCA) precipitates by the centrifuge method^31^,^32^. For each sample, triplicate aliquots (1.5 mL) and one TCA-fixed control were incubated with 10 nmol l^−1^ of ^3^H-leucine for 2 h at *in situ* temperature in the dark. The incorporation was stopped with the addition of TCA (final concentration, 5%). Samples were centrifuged, aspired, and washed with 5% TCA. After addition of scintillation cocktail, samples were radioassayed.

### Flow-Cytometric analyses

Abundances of heterotrophic prokaryotes, small non-pigmented flagellates and pigmented eukaryote populations all were quantified using a Beckton-Dickinson FACSCalibur flow cytometer equipped with a blue (488 nm) argon laser set at 15 mV. All parameters were collected as logarithmic signals obtained from the machine standard filters and detectors: the green fluorescence of the DNA stain SybrGreen I (Molecular Probes, Invitrogen) and the orange (phycoerythrin) and red (chlorophyll) fluorescences were collected in the FL1, FL2 and FL3 channels, respectively^33^. Flow rate calibration was performed daily for abundance estimation, and yellow-green fluorescent latex beads (0.92 μm, Polysciences [10^6^ mL^−1^]) were used as internal standards. In each analyzed sample of heterotrophic bacteria and picophytoplankton, beads were added at final concentrations of cs. 10^5^ and 10^4^ mL^−1^, respectively. Populations were delimited and abundances were estimated with the softwares CellQuest and PaintAGate (BectonDickinson, Palo Alto, CA).

Abundances of small (<5 μm) pigmented eukaryotes were determined *in vivo*, a few minutes after sampling. A volume of 0.5 mL of each sample was run at high speed (50–80 μl min^−1^) for 3 minutes and populations were identified in a SSC (side scatter)-FL3 (red fluorescence) and a FL2 (orange fluorescence)-FL3 dot plots. Three populations (*Synechococcus* and two size fractions of pigmented eukaryotes) were separated according to size and type and level of fluorescence. Since the last two groups exhibited highly similar temporal dynamics, they were treated as one homogenous group in the data analysis (phototrophic eukaryotes, PE). Conversion of their relative side scatter signal^34^ yielded an average cell diameter for this group of 1.7 μm (range 1.3–2.2 μm; Fig. S1).

Total heterotrophic bacteria abundances were obtained from 1.4 mL samples preserved with 1% paraformaldehyde +0.05 mL glutaraldehyde solution (final concentration) on a 1:10 (v:v) dilution that was kept for 10 min at 4 °C in the dark, deep frozen in liquid nitrogen, and stored at −80 °C until further analysis. Prior to the analyses, which were conducted within 1 or 2 days of sampling, the samples were thawed and stained with a 10x dilution SybrGreen I (Molecular Probes) solution (final dilution, 1:1,000 (v:v)), kept in the dark for 15–20 minutes, and run at low speed (~20 μl min^−1^) for 90 seconds. Strictly, this analysis quantified all non-phototrophic prokaryotes, comprising chemo-autotrophic and chemo-heterotrophic archaea and bacteria. However, chemoautotrophs and archaea are generally low in abundance in the surface waters of the Eastern Mediterranean^35^. We therefore refer to this group as heterotrophic bacteria (HB) for convenience.

Heterotrophic flagellates (HF) were determined from 4.5 mL samples fixed with 1% glutaraldehyde (final concentration), deep frozen in liquid nitrogen and stored at −80 °C until analysis. Within a few months, 2 mL samples were stained with a 1:10,000 SybrGreen I (final concentration), for 10 min in the dark, run at high speed (82 μl min^−1^) for 5–6 minutes; HF were detected flow cytometrically with SSC vs FL1 and FL1 vs FL3 dot plots [see^36^ for more details]. No beads were added for this analysis.

### Data analysis

To test for linear vs. unimodal trends along the light gradient, we conducted linear regression analysis with light as a log-transformed and squared log-transformed variable. Model selection (linear *vs* unimodal) was performed based on the Akaike Information Criterion^37^. We analyzed potential interactions between bacteria and PE by regressing the net-growth rates of bacteria against the corresponding abundances of PE and HF within a given light treatment.

## Results

### Light manipulation

The application of neutral density filters resulted in a logarithmic light-gradient, with an average light intensity (*I*_*mix*_) of 40, 22, 12 and 4.5% relative to the incoming light (Fig. 1; the average light intensities differ slightly from those given in^26^ due to an error in the previous calculation). According to the intensity of the filter foils, we refer to these levels as L95 (transmission = 95%, highest light level) to L12 (transmission = 12%, lowest light level).

One mesocosm bag (L95 B) started losing water after 2 days. Due to the differences observed in water salinity in the mesocosms (ca. 40 ppm) and the surrounding water (<20 ppm), this mesocosm soon became drop-shaped, resulting in reduced light penetration. It was excluded from all analyses.

### Dissolved nutrients

Regular measurement by standard methods yielded concentrations below detection limit for phosphate throughout the experiment (detection limit = app. 0.02 μmol P L^−1^). Concentrations of NO_3_ and NH_4_ were near or below detection limits in most samples. Averaged over the last three samplings (days 4, 6, 8), NO_3_ and NH_4_ decreased significantly with increasing light (NO_3_: p = 0.03, r^2^ = 0.54; NH_4_: p < 0.01, r^2^ = 0.75; linear regression with light ln-transformed).

### Phosphorus-uptake

**Figure 2:**
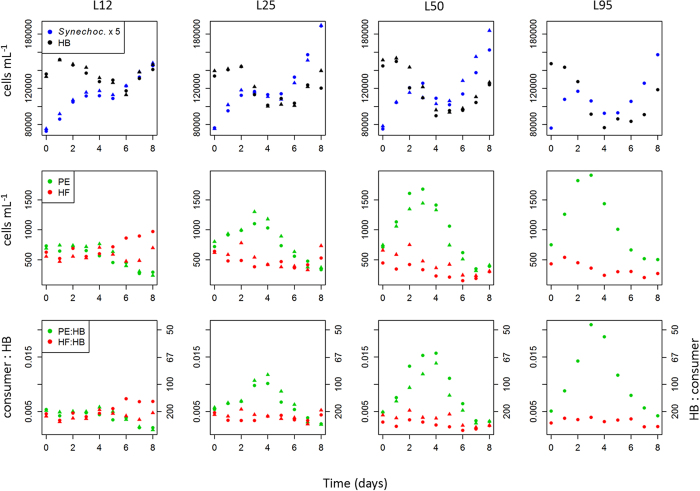
Upper row: time course of heterotrophic bacteria (HB) and *Synechococcus*. Note that abundances of *Synechococcus* are multiplied by 5 to scale them with HB. Lower row: Ratio of photosynthetic picoeukaryotes PE:HB and HF:HB, based on cell concentrations, as an indicator of potential grazing control of flagellates on HB (cf. Gasol 1994). The inverse ratio is given on the 2^nd^ y axis. Figure symbols distinguish replicates.

As revealed by size-fractionated radio-assay analysis of ^33^P uptake, the two smallest fractions (0.2−<0.6 μm and 0.6–2 μm) accounted together for almost 90% of P-uptake (Tanaka, unpub. data). Given the numeric dominance of HB and *Synechococcus* over PE (*Synechococcus* exceeded PE by a factor of 20–30; Fig. 2), this clearly suggests that these prokaryotic groups dominated P-uptake in the experimental communities.

### Prokaryotes

**Figure 3:**
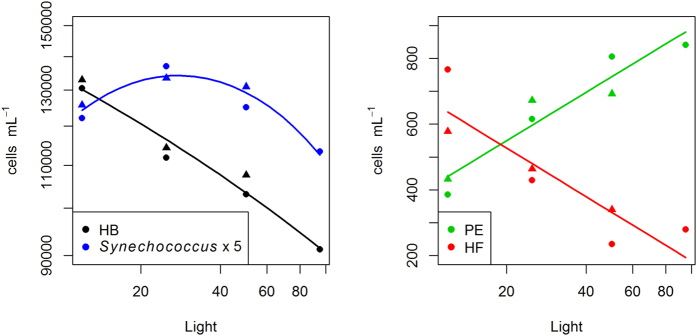
Average abundances of HB & *Synechococcus* (left) and PE & HF (right) along the light gradient (avg. days 4–8). Note that abundances of *Synechococcus* are up-scaled by a factor of five to align them with HB. All regression lines are highly significant. Summary statistics are given in Table S1.

Picoplankton were numerically dominated by heterotrophic bacteria (HB) and *Synechococcus* (Syn), whereas abundances of *Prochlorococcus* were very low (<1% of total prokaryotes). HB and *Synechococcus* exhibited similar dynamics over time, with a first increase and peak around days 2–4, followed by a drop and a second increase over the last two days (Fig. 2). Averaged over days 4–8, both *Synechococcus* and HB decreased with increasing light (Fig. 3). However, while HB peaked at the lowest light level (L12), *Synechococcus* peaked at L25.

### Small eukaryotes

The most abundant eukaryotic group was PE, with abundances ranging from 300 to 1500 cell mL^−1^ and were thus in a similar range as reported earlier for the Eastern Mediterranean^38^,^39^. Abundances of heterotrophic flagellates (HF) were somewhat lower, ranging from 150–950 cells mL^−1^. Across all treatments, abundances of HF and PE exhibited a negative correlation on all days, which was significant on days three, four, five and eight (data not shown, but compare with the averages of day 4–8, which are shown in Fig. 3).

### Bacterial activity

Similar to the abundances of heterotrophic bacteria, bacterial activity was inversely related to light intensity. When scaled to specific activity per cell, our estimates of ‘intrinsic growth’ were independent from light (Fig. S2).

### Grazing-control of bacteria

As an indication for potential grazing control of HB by PE or HF, we calculated the ratio of prey with their putative consumers (Fig. 2). PE:HB exhibited an inverse trend over time compared to HB abundances and showed the most pronounced peak at L95, while the ratio HF:HB was rather flat with time (Fig. 2).

**Figure 4:**
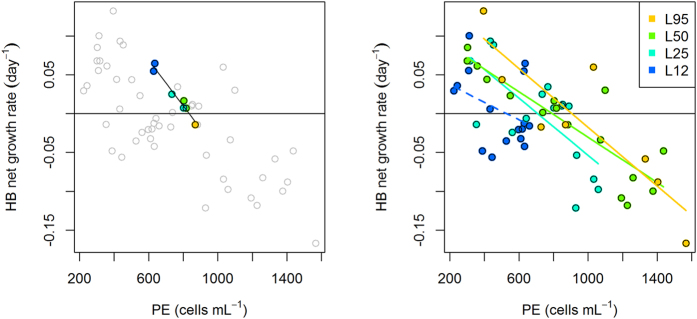
Net growth rates of heterotrophic bacteria (HB) plotted against abundances of PE. Growth rates were calculated for adjacent time intervals, PE abundances averaged for the corresponding time intervals. Left plot: The colored points give the relationship for the first time interval (day 0–day 1), together with a linear fit (*p* < 0.001). Right plot: Growth rates for all time intervals. Linear fits calculated separately for each light level. *p* < 0.001 except for L12 (*p* = 0.1). Correlation tests for the same data are given in Table 1.

In order to perform a more direct test for potential top-down effect exerted by PE and HF on HB, we calculated the net growth rates of heterotrophic bacteria for each sampling interval and mesocosm. Assuming that bacterial activity was independent from light (see above), we related HB net growth rate to the abundances of the putative bacterivores, PE and HF. For the first sampling interval (day 0-day 1), when conditions in terms of nutrient concentrations and abundances of larger consumers (micro- and mesozooplankton) were most comparable among light levels, we performed a test across all light levels (Fig. 4left). At this time point, PE showed a moderate increase with light (600 to 800 cells mL; averages Day 0 & Day 1). In spite of this moderate variation in PE abundances, HB net growth rates exhibited a consistent linear negative relationship with abundances of PE (Fig. 4left) (while no additional relationship with light was detected when regressing HB net growth against both parameters in a multiple linear regression). Conversely, HB net growth did not relate to HF abundances for this sampling interval (data not shown). In a second analysis, we regressed HB net growth rates from all time points against the corresponding abundances of PE and HF within each light level. This analysis again revealed significant negative relationships between bacterial growth rates and PE abundances in all light levels except for L12 (Fig. 4right). Conversely, for heterotrophic flagellates, relationships between HF and HB growth rates were much weaker and significant only for L50 (Table 1). For *Synechococcus*, we performed only the second analysis within light levels, as *Synechococcus* growth should scale with light intensity. Net growth rates of *Synechococcus* were clearly negatively correlated with PE, but not with HF (Table 1).

HF abundances decreased over time in all treatments except for the lowest light level, where a moderate increase was observed (Fig. 2; Spearman’s rho correlation for HF vs day of the experiment. L12: 0.52 (p = 0.025, n = 18); L25: −0.35 (p = 0.15, n = 18); L50: −0.7 (p = 0.001, n = 18); L95: −0.83 (p = 0.005, n = 9)).

## Discussion

Following our expectations, the components of the small plankton showed a fast response, as visible by the opposing time trends seen among HB and *Synechococcus* on the one hand, and PE on the other (Fig. 2). In line with this idea, these groups exhibited significant responses with regard to the light treatments (Fig. 3).

Concentrations of dissolved phosphorus were below the detection limit (0.02 μmol L^−1^) throughout the experiment, in line with the generally acknowledged P-deficiency of Eastern Mediterranean waters^40^,^41^.

One mesocosm bag, L95B, was excluded from the analysis due to water leakage; hence we lack a replicate in the high-light treatment. Given the other treatments showed consistent replicates, and because L95A fits well into the overall pattern observed, we believe it is valid to consider this treatment in the overall discussion of the data.

Our interpretation of PE being the predominant bacterivores in our experiment is indirect, as we lack direct observations on bacterivory. Overall, the ratio PE to HB (cell concentration; 0.005–0.015, Fig. 2) was rather high. Gasol^42^ suggested that a bacteria:bacterivore ratio of <200 (corresponding to >0.005 bacterivore:HB in our case) would indicate top-down control of the prokaryotes. Assuming that PE contained a large fraction of bacterivorous flagellates, the ratios reported here (maximum 0.02 in L95) suggest strong top-down control of HB by PE. Conversely, the HF:HB ratio was generally lower than the PE:HB ratio in all treatments except L12 where PE:HB and HF:HB ratios were similar. PE and HB exhibited a pattern resembling predator-prey oscillations. The numeric response of PE peaked at the highest light level, suggesting a direct positive light effect on PE growth. This peak abundance in PE coincided with the strongest drop in abundances of HB and *Synechococcus* in the same treatments (L95; Fig. 2). The notable increase of HB and *Synechococcus* after day 4 coincided with a decrease of PE. This reversed pattern towards the end of the experiment may reflect a cascading effect exerted by larger protistan grazers, which peaked towards the end of the experiment^26^. Across the experiment, the direct interaction between PE and HB is generally supported by the consistent negative relationship between HB net growth rate and PE abundances (Fig. 4, Table 1), plus from the fact that specific bacterial activity was independent from light. Taken together, these patterns indicate light-driven bacterivory by PE. The same explanation also matches the temporal dynamic seen in *Synechococcus*. In line with this, cell diameter of PE clearly exceeded that of *Synechococcus* (Fig. S1). However, in contrast to HB, we cannot assess the underlying gross growth rates in *Synechococcus*.

### Mixotrophy in small phototrophic eukaryotes (PE)

Our observations are congruent with the emerging knowledge on the role of small mixotrophic protists in the food web of oligotrophic marine sites. According to recent field studies, the PE community is composed of various phylogenetic groups. Besides typical mixotrophic representatives like prymnesiophytes, chrysophytes and cryptophytes, also prasinophytes of the group mamiellales, which are believed not to be mixotrophic, may contribute a substantial fraction of the PE community (e.g. ref. 43). However, recent studies show that mamiellales are especially abundant in relatively nutrient rich waters, like coastal and arctic waters, while they are rare in nutrient depleted oceanic waters, where especially prymnesiophytes and chrysophytes are highly abundant^10^,^44^. Exhibiting year-round nutrient limitation and low chlorophyll levels, the Eastern Mediterranean compares much more to an oceanic than to a coastal environment. A recent survey that specifically addressed the composition of the PE in the Eastern Mediterranean indeed reported that Prymnesiophyceae, Pelagophyceae, Chrysophyceae and Cryptophyceae were the most abundant groups within the PE fraction^45^. Similarly, potentially mixotrophic groups dominated PE composition in the Gulf of Naples^46^, while mamiellales were rare in both studies. Microscopic analysis did not allow a quantitative analysis of the smallest fractions that were counted as PE by flow cytometry; among the phytoplankton that could be identified microscopically, the most abundant group were small haptophytes similar to *Imantonia sp* (A.F. Sazhin, unpub. data). It is thus conceivable that the majority of PEs in our samples belonged to the groups that have been shown to be mixotrophic. Chlorophytes were never observed to be mixotrophic in studies in the Mediterranean Sea^47^ even though a recent study reports bacterivory in an arctic-isolated strain of *Micromonas*^48^.

### Assessment of hypothesis 1

We expected an inverse relationship between mixotrophic and heterotrophic bacterivores along the light gradient. This hypothesis was clearly confirmed (Fig. 3). In fact, the apparent role of HF was overall weak in this experiment, as seen by the weak relationships between bacterial growth rates and HF abundances (Table 1). The negative time trend of HF in all except the lowest light treatment further suggests that HF were inferior competitors at preying upon HB and *Synechococcus*. Only towards the end of the experiment HF started to increase in L12 and L25. This may be related to above mentioned cascading effects evoked by lager protistan grazers at this time (concomitant decrease of PE and increase of HB, Fig. 2), as HF may achieve very high growth rates at saturating prey levels^18^,^49^.

### Assessment of hypothesis 2

We expected that mixotrophic bacterivores would exert a light-dependent mortality pressure on HB. The analyses of bacterial net- and gross growth rates (as approximated by leucine incorporation), together with their final abundance suggest such a relationship. Viral abundances, on the other hand, consistently showed a positive relationship with abundances of HB, hence viral control does not appear plausible (Gomes *et al*. in prep). Similar to HB, *Synechococcus* also exhibited an overall negative trend with light, with peak abundance at the second lowest light level. As *Synechococcus* itself depends on light, this pattern could reflect that these organisms were top-down controlled by PE at L95 & L50, but were light limited at L12. Assuming an average daytime light intensity of 600 μE m^−2^ s^−1^ (end of September at 34°North), the average light intensity in the L12 treatment was near 25 μE, which is well below the saturating light level for *Synechococcus* (e.g. ref. 50). At the same time, the cell-specific auto-fluorescence for phycoerythrine was very high in the L12 treatment, indicating light-limitation in *Synechococcus* (Royer *et al*. in prep).

### Stoichiometric implications of heterotrophic vs mixotrophic bacterivory

Cellular C:N and especially C:P ratios of heterotrophic bacteria are low compared to the elemental composition of protists^15^. The gross growth efficiency of heterotrophic consumers typically is in the range 30–50%, meaning that they respire 50–70% of the organic carbon assimilated from their prey^49^. Due to the low C:P ratio of heterotrophic bacteria, bacterivorous consumers face a particular stoichiometric mismatch between their nutritional demands and the elemental composition of their prey^23^,^51^. As a result, heterotrophic bacterivorous flagellates and ciliates effectively remineralize a considerable amount of the nitrogen and phosphorus ingested with their prey, and they play a key role in microbial food webs in shunting nutrients from bacteria back to the dissolved phase and thus making them available to the phytoplankton^52^.

To understand the ecosystem consequences of mixotrophic bacterivory, we need a better quantitative understanding of their metabolic pathways. This is presently lacking. Available studies show that the organic carbon ingested with prey cannot fully supplement energy demands in many bacterivorous mixotrophs, as phagotrophy allows only for limited or no growth in the absence of light^53^,^54^,^55^,^56^,^57^. Moreover it appears from numerous experiments (e.g. ref. 53,57, 58, 59) that many bacterivorous mixotrophs ingest prey primarily to supplement their requirement in mineral nutrients by phagotrophy. This matches with the inverse pattern of PE and their prey along the light gradient in our experiment. That is, instead of shunting dissolved nutrients from remineralized prey back into the dissolved phase, mixotrophs utilize nutrients from bacterivory directly for photosynthesis, thereby representing a light-induced shortcut in the microbial loop.

The dominant role of PE as bacterivores deduced from our experiment is in agreement with a large number of recent studies^5^,^6^,^7^,^14^,^60^. The novelty of our study is in demonstrating that the impact of mixotrophic bacterivores is light-dependent. A light-induced shift in the predominant type of bacterivory has fundamental implications for nutrient dynamics. First, as mixotrophs apparently do not act as net-remineralizers under light-saturating conditions^23^, we should expect that mixotrophic grazing affects nutrient turnover rates with direct consequences for (other) photoautotrophic phytoplankton. Second, as shown in our experiment, the light-induced bacterivory by mixotrophic picoeukaryotes results in reduced abundances of HB and *Synechococcus*. A numeric reduction of HB and *Synechococcus* very likely has direct implications for food web efficiency in this environment where conversion of dissolved nutrients into new biomass relies primarily on these organisms.

## Conclusions

Our data strongly indicate that light mediates bacterivory by selectively favoring mixotrophic pico-grazers. The results point out that in oligotrophic systems, light should be considered as a key variable affecting the dynamics of microbial food webs.

The recent appreciation of a prevalent role of mixotrophic bacterivores in oceanic surface waters challenges the classical view of the microbial loop regarding the role of bacterivores for nutrient cycling. The missing representation of mixotrophy in models of the microbial food web may cause serious biases for our understanding of nutrient cycling and trophic dynamics^61^,^62^. Empirical data on the light-dependent kinetics of mixotrophic bacterivory are urgently needed to enable a realistic representation of their functional role in microbial food webs.

## Additional Information

**How to cite this article**: Ptacnik, R. *et al*. A light-induced shortcut in the planktonic microbial loop. *Sci. Rep*. **6**, 29286; doi: 10.1038/srep29286 (2016).

## Supplementary Material

Supplementary Information

## Figures and Tables

**Table 1:** Correlation tests between bacterial net growth rates (HB = heterotrophic bacteria, *Syn* = *Synechococcus*) and abundances of bacterivores (phototrophic eukaryotes (PE) and heterotrophic flagellates (HF)).

Light	Phototrophic eukaryotes		Heterotrophic flagellates	
	HB	*Syn*	HB	*Syn*
L95	−0.83 (**0.015**)	−0.81 (**0.022**)	−0.69 (0.055)	−0.17 (0.69)
L50	−0.87 (**<0.001**)	−0.70 (**0.004**)	**−0.57 (0.023)**	−0.04 (0.874)
L25	−0.65 (**0.008**)	−0.74 (**0.001**)	−0.18 (0.512)	0.16 (0.556)
L12	−0.17 (0.534)	0.24 (0.361)	−0.19 (0.491)	−0.43 (0.096)

The test was performed for all light levels separately. Given is Spearman’s rho together with the corresponding p-value in brackets. *n* = 7 for all tests.
