# Supplementary material for: A light-induced shortcut in the planktonic microbial loop
Source: Sci Rep. 2016 Jul 11;6:29286. doi: 10.1038/srep29286 (PMC4941531; doi:10.1038/srep29286)

A light-induced shortcut in the planktonic microbial loop

Robert Ptacnik*, Ana Gomes, Sarah-Jeanne Royer, Stella A. Berger, Albert Calbet, Jens C. Nejstgaard, Josep M. Gasol, Stamatina Isari, Stefanie D. Moorthi, Radka Ptacnikova, Maren Striebel, Andrey F. Sazhin, Tatiana M. Tsagaraki, Soultana Zervoudaki, Kristi Altoja, Panagiotis D. Dimitriou, Peeter Laas, Ayse Gazihan Akoglu. Rodrigo A. Martínez, Stefanie Schabhüttl, Ioulia Santi, Despoina Sousoni & Paraskevi Pitta

Supplementary data

Table S1: Regression statistics for Figure 3. *n*=7 for all tests.

		Estimate (significance level)	
	Adj. rsq.	ln(light)	$\ln(\text{light})^2$
HB	0.95	-18688 (p<0.001)	n.s.
Synechococcus	0.85	19008 (p=0.005)	-2862 (p=0.005)
PE	0.89	212 (p<0.001)	n.s.
HF	0.77	-214 (p=0.006)	n.s.

Fig. S1. Cell diameter distribution (in µm) of *Synechococcus* and PE as estimated from the relative side scatter signal (Calvo-Díaz & Morán 2006).

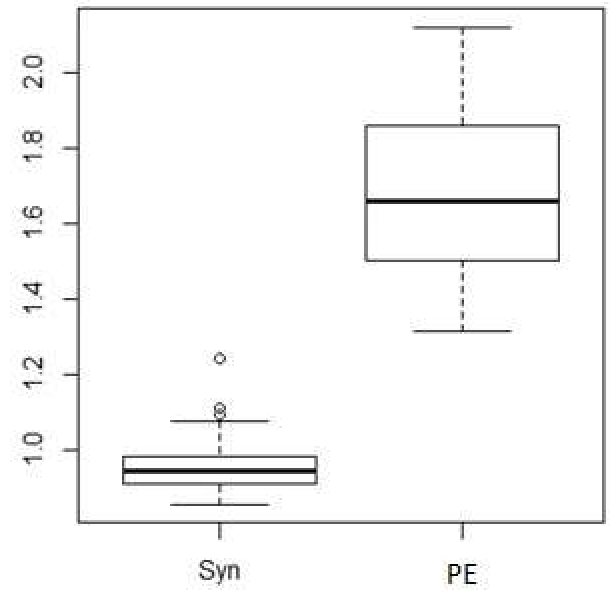

Fig. S2. HB activity related to HB abundances (left) and ln(light) intensity (right) in a multiple linear regression. Shown are the partial residuals together with mean (solid line) and confidence interval (broken line).

($p<0.001$ for HB and $p=0.96$ for ln(light); adj.rsq=0.41; df=69.)

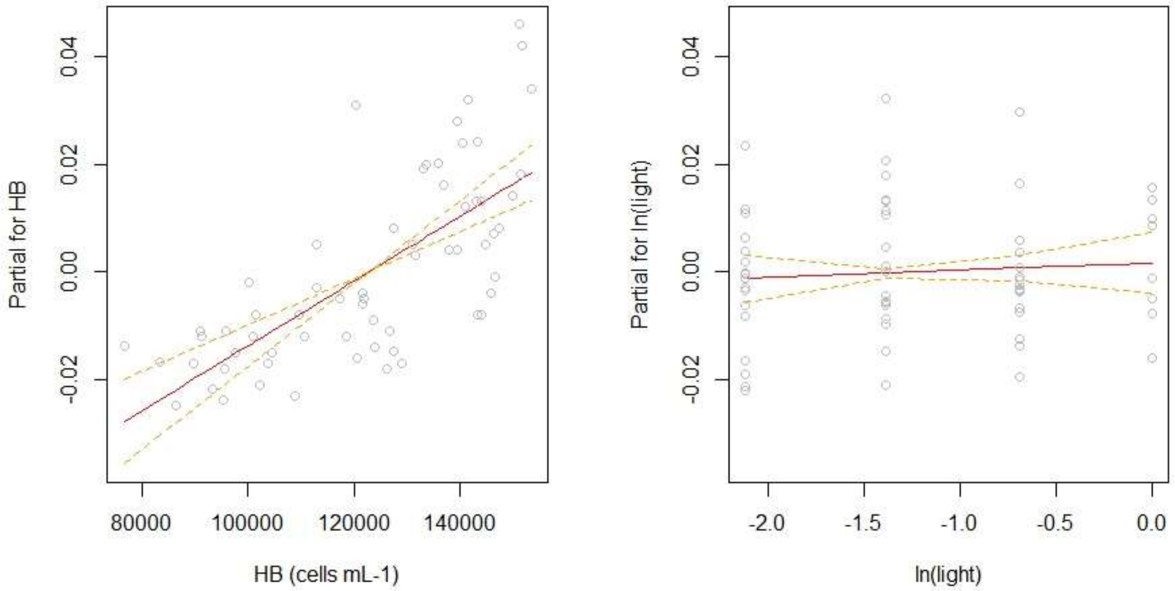

Supplement: Supplementary Information [file srep29286-s1.pdf]
